# Acute effects of reducing sitting time in adolescents: a randomized cross-over study

**DOI:** 10.1186/s12889-017-4660-6

**Published:** 2017-08-15

**Authors:** Anisse Penning, Anthony D. Okely, Stewart G. Trost, Jo Salmon, Dylan P. Cliff, Marijka Batterham, Steven Howard, Anne-Maree Parrish

**Affiliations:** 1 Faculty of Social Sciences, University of Wollongong, Wollongong, NSW 2521 Australia; 2 Mathematics and Applied Statistics, University of Wollongong, Wollongong, NSW 2521 Australia; 3 Early Start Research Institute, University of Wollongong, Wollongong, NSW 2521 Australia; 4 Institute of Health and Biomedical Innovation at Queensland Centre for Children’s Health Research, Queensland University of Technology, South Brisbane, QLD 4101 Australia; 5 Centre for Physical Activity and Nutrition Research, Deakin University, Melbourne, VIC 3125 Australia

**Keywords:** Sedentary behaviour, School, Cardiometabolic, Executive function, Youth

## Abstract

**Background:**

Levels of sitting among adolescents are high, especially during the school day. The acute cognitive and health consequences associated with prolonged sitting are poorly understood in adolescents. This randomized crossover design study examined the acute effects of a simulated school day with reduced sitting or usual sitting on adolescents’ cognitive function and cardiometabolic biomarkers.

**Methods:**

Eighteen healthy school aged adolescents were recruited from the community to the study (11 males; 7 females; mean age [SD] = 13.5 ± 0.9 years). Two protocols were developed to simulate an adolescent school day, the amount of time spent sitting was manipulated reflecting: a ‘typical’ day (65% of the time spent sitting with two sitting bouts sitting >20 min) and a ‘reduced sitting’ day (adolescents sat for 50% less time with no bouts of sitting >20 mins). The order that participants were exposed to each condition was randomized (via random number generator).

Participants were not fully blinded as they could observe the difference between conditions. Energy intake and moderate to vigorous physical activity (MVPA) were standardized for both conditions and monitored for 48 h post-condition for compensatory effects. Cognitive (working memory) and cardiometabolic outcomes (lipids, glucose, insulin, IL-6, apo-A1, apo-B, blood pressure,) were assessed pre and post for both conditions, BMI and body fat were assessed on the morning of the intervention. Data were analyzed using linear mixed models. Standardised effect sizes were calculated.

**Results:**

Compared with the typical school day, the reduced sitting day demonstrated significant positive effects for apoB/apoA-1 ratio (adjusted difference ± SD) -0.02 ± 0.03; *P* = 0.03; effect size [Cohen’s *d*] = −0.67. Findings for total cholesterol −0.19 ± 0.27; *P* = 0.28; *d* = −0.71; HDL cholesterol −0.23 ± 0.50; *P* = 0.12 *d* = −0.66; and total cholesterol/HDL ratio 0.25 ± 0.53; *P* = 0.25; *d* = 0.51 and for cognition 0.64 ± 0.15; *P* = 0.15; *d* = 0.54 were non-significant. There were no compensatory changes in participant energy expenditure or energy intake for 48 h post intervention.

**Conclusion:**

Reducing school day sitting time in adolescents’ resulted in significant improvements in apoB/apoA-1 ratio with medium effect sizes for total cholesterol, HDL cholesterol and total cholesterol/HDL ratio. Cognitive function results showed the equivalent of a 6 month improvement in effective mental-attentional capacity.

**Trial registration:**

The trial was registered as a clinical trial with the Australian and New Zealand Clinical Trials Registry (ACTRN12614001064695) on the 3rd of October 2014 - registered retrospectively.

**Electronic supplementary material:**

The online version of this article (doi:10.1186/s12889-017-4660-6) contains supplementary material, which is available to authorized users.

## Background

Adolescents have particularly high levels of sedentary behavior with breaks in sedentary time markedly decreasing as they age, making them vulnerable to possible adverse health effects [11]. Current literature investigating the effects of sedentary behavior on cardiometabolic markers in adolescents is limited with mixed results. A crossover trial of non-overweight adolescents fed a healthy meal while undergoing three experimental conditions to assess the impact of reduced sitting time on cardiometabolic outcomes produced no measurable changes in cardiometabolic markers [46]. An experimental trial involving 10 to 14 year old children who consumed a high fat meal, interrupting sitting time with bouts of moderate to vigorous physical activity (MVPA) resulted in a reduction in triglyceride concentrations [43]. Another trial reported that interrupting sitting with MVPA reduced insulin and free fatty acid concentrations in children aged 7–11 years [6]. Few studies have investigated the impact of breaking up sitting with light physical activity (LPA) breaks on cardiometabolic outcomes. One trial assessed the impact of a LPA treatment compared to a sitting day in 10 to 18 year olds and reported observing significant treatment by time interactions in HDL cholesterol and insulin, however the treatment effect was not significant [48]. Collectively these studies suggest that reductions in sitting time are associated with positive health outcomes in adolescents but more research is needed.

Adolescent spent large portions of their day sitting in controlled environments such as schools [9]. Thus, experimental studies conducted in school setting may be more generalizable to large proportions of the adolescent population. Adolescents have fewer breaks in sedentary time during the school day than in any other period of the week [30]. To date, experimental studies have been conducted under controlled conditions, often testing the effect of a high-fat or high-carbohydrate meal on energy expenditure. As such, they are not indicative of a balanced dietary intake. There are no experimental studies which replicate a school day.

There is also growing interest in the effects of prolonged sitting on cognitive outcomes [54]. Particular interest has been directed toward working memory, a core ‘executive function’ for activating, maintaining and manipulating information in the mind given its relation to academic achievement [1], learning, reasoning, and cognitive control [28]. Initial evidence suggests that breaking up sedentary time may improve cognitive outcomes in children [16]. However there is a dearth of literature assessing the effect of reducing and breaking up bouts of sitting time in the school setting. An understanding of the effects of sitting time on both cognitive and health outcomes in the classroom setting could inform future classroom interventions with potential positive long term effects for children.

This study aimed to examine the acute effects of reducing total and prolonged bouts of sitting time compared to a traditional sitting time during an adolescents’ school day on cognitive function and cardiometabolic outcomes in a laboratory setting. It was hypothesised that there would be more favorable changes in cognitive function and cardiometabolic health outcomes following a simulated “reduced sitting” school day when compared to a simulated typical day and that participants would not compensate by altering their energy expenditure or intake. This intervention is particularly novel in that it replicated a typical school day and thus holds potential for translation to the school setting in future interventions.

## Methods

### Participants

Adolescents were recruited from the Illawarra region of New South Wales (population 0.4 M) via a university media release, university website, local newspaper, radio station, high school newsletters and referrals from local pediatricians between May and July 2014. Parents and guardians of interested participants were interviewed over the telephone to ascertain if their adolescent was eligible for the study. Adolescents were excluded if they were: outside the predetermined age range (12–15 years), taking one of the exclusionary medications (i.e. Ritalin, long- term steroids or anti-psychotic medications) or had one of a pre-determined list of conditions or disabilities (i.e. Prader-Willi syndrome, Bardet-Biedl syndrome, Type 2 Diabetes, coeliac disease, PKU or other metabolic disorders, cystic fibrosis, multiple food allergies and a significant physical disability or developmental disability which may inhibit participation). Ethics approval was gained from the University of Wollongong Human Research Ethics Committee (HE14/069). Informed written parental and participant consent was gained for each participant.

### Study design

This study used a randomized cross-over design. The trial is reported in accordance with the CONSORT statement [37] and was registered as a clinical trial with the Australian and New Zealand Clinical Trials Registry (ACTRN12614001064695).

### Study protocol

**Fig. 1:**
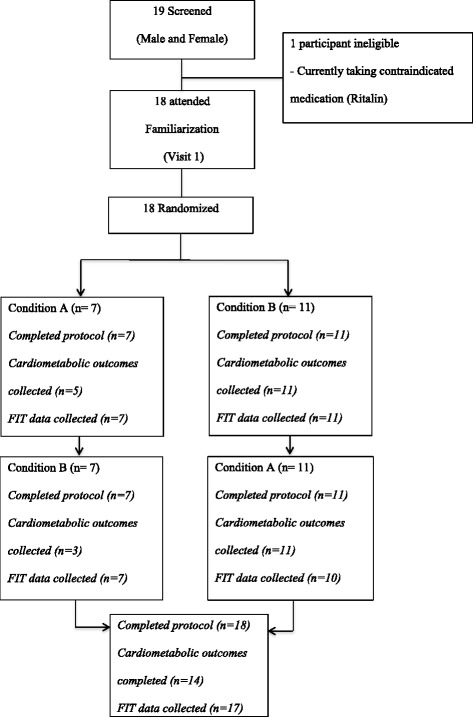
*Blood was unable to be drawn from two participants post ‘Visit 2’. They therefore did not participate in blood collection during ‘Visit 3’

Participants were instructed not to restrict their food or normal activities during the day prior to the experiment, however they were required to fast for 8 h overnight before arriving [2, 32] at the University at 08:00 a.m. Participants visited a laboratory-style setting at the university on three occasions (Fig. 1-flow diagram). The laboratory accommodated two participants at one time in separate rooms. Visit 1 involved the parent/guardian and adolescent becoming familiarised with the facility. Visits 2 and 3 were the randomized test conditions, either the ‘typical’ school day (Additional file 1), or the ‘reduced sitting’ school day (Additional file 2) (Visit 3 was identical to Visit 2 except that participants completed the condition not undertaken in Visit 2). Both protocols were designed to simulate an adolescent school day reflecting typical high school timetabling and competencies. The amount of time spent sitting was manipulated to simulate the two conditions, a ‘typical’ school day (where adolescents spent 65% of the time sitting and engaged in two bouts of sitting >20 min (mins) [9] and a ‘reduced sitting’ school day (where adolescents sat for 50% less time with no bouts of sitting >20 mins). Efforts were made to schedule Visits 2 and 3, 1 week apart and if possible on the same day of each week. In total, participants spent the duration of an average school day (6 h) completing the protocol for both Visits 2 and 3.

### Procedure

Prior to commencing the protocol anthropometric measures were assessed using standardized protocols, the measures included: height, weight, body composition (weight, body fat percentage and BMI), resting heart rate and blood pressure. Height was measured using a portable stadiometer, according to standardized procedures whilst weight and body composition was assessed using a segmental body composition analyzer (Tanita BC-418A, Tanita Corporation, Illinois, USA). Weight status was calculated according to the participants’ body fat percentage based on the Child Growth Foundation centiles [34]. Participants were required to sit for a minimum of 5 min before having their resting blood pressure and heart rate measured by an automatic blood pressure monitor (Omron model IA1B, Healthcare Australia). Subsequently a registered nurse/phlebotomist collected approximately 10-ml of the participant’s blood for analysis.

Participants were then given a choice of food items to eat during the visits. They were informed that their food selections were not restricted; however, they had to consume the same amount and types of food during both Visits 2 and 3. They chose from a variety of breakfast, mid-morning snack and lunch options (e.g. cereal, fruit, muffins and sandwiches), each individuals chosen items were recorded. They consumed breakfast prior to commencing the protocol. The mid-morning snack was consumed 2 h and lunch 4 h after the commencement of the protocol.

The protocols commenced (9 a.m.) and ended (3 p.m.) at approximately the same time of day for all participants (Additional files 1 and 2). Participants progressed through the activities on the protocol directed by a trained research assistant to ensure efficacy. At the commencement of the both protocols the participants completed the paper based measure of mental attention capacity, (Figural Intersectoral Task - described in detail below). The protocol incorporated time in homeroom (or roll call), class time (e.g. Maths, English, Physical Education, History and Art), walking between classes and recess/lunch breaks for 370 min. The ‘typical’ school day protocol consisted of 240 min of sitting time, 102 min of LPA and 28 min of MVPA (Additional file 3). The ‘reduced’ school day protocol consisted of 117 min of sitting time, 225 min of LPA and 28 min of MVPA. This Figural Intersectoral task was repeated just prior to the end of the school day protocol. When the protocol had ended the same anthroprometric measures were collected, using the methods adopted prior to commencing the protocol.

### Typical school day

The protocol for the amount of time spent sitting or being physically active during the ‘typical’ school day (Additional file 1) was informed by a previous study which reported adolescents sat for an average of 240 mins during a typical school day spending 102 mins in LPA [9]; the remaining 28 min of the school day protocol spent in MVPA (Additional file 3). The development of the protocol required each activity in the protocol being matched to the appropriate level of physical activity intensity (e.g. sitting (sedentary), light-intensity physical activity (LPA) or moderate-to-vigorous-intensity physical activity (MVPA) [42]. During the experiment participants simulated walking between classes to their next class with the use of a treadmill at low speeds (1.5–2.0 km/h). The time spent in MVPA was constant for both conditions, and simulated by participants walking or running on a treadmill at assigned times during the protocol for a total of 28 mins (5.0–6.0 km/h).

### Reduced sitting school day

The protocol for the ‘reduced sitting’ school day (Additional file 2) was similar to the procedures adopted for the typical school day, except that it involved approximately 50% less sitting time than the protocol for the ‘traditional’ school day. In the reduced school day protocol time previously spent sitting was replaced with LPA. To achieve this goal, protocol activities were modified to include an adjustable standing desk and regularly breaks in sitting time (e.g. maximum 20 mins sitting time) with investigator led standing and stretching or other LPA activities (for 2–4 min). Participants spent a total of 28 mins in MVPA as per the traditional protocol.

### Outcome measures

#### Mental attention capacity

During both Visits 2 and 3 the participants completed the Figural Intersections Task (FIT) at the start and end of each condition [40]. The FIT is a paper-based measure of mental attentional capacity – (a causal factor underlying developmental growth of working memory). Participants were required to locate an area of common intersection among two to eight overlapping shapes. The number of shapes that must simultaneously be manipulated in mind corresponds to the item’s mental attentional demand. The task consisted of 36 items of differing item difficulty. There were five items at each level of difficulty (item class) with the exception of class 4 which had one additional item. Each item began by participants placing a dot inside each discrete shape on the right side of the page and then one dot in an area of common intersection of those shapes on the left side of the page. Seven items incorporated irrelevant shapes, in which case participants had to ignore the extra shape when identifying the area of common intersection. These items were considered as one class higher for the purposes of scoring. Training (i.e., instructions and 8 practice items) was provided before commencement of the task. The task took approximately 40 mins to complete. Performance on the FIT was indexed by a FIT *k* score, which corresponds to the highest item class with at least 80% accuracy, provided all lower item classes also meet or exceed this threshold (with one lower item class permitted to fall to 60% accuracy).

#### Cardiometabolic health indicators

The selection of the cardiometabolic indicators for this study was determined firstly by consulting previous literature where triglycerides, HDL cholesterol, glucose, insulin, systolic/diastolic blood pressure were identified [10, 12, 17, 19, 32, 44, 47], in addition to more novel markers ApoB and ApoA-1 [8] and Interleukin-6 (IL-6) [3].

Blood draws were taken at pre-intervention following an overnight fast and post-intervention, 2.5 h after lunch. The blood was analyzed for 10 cardiometabolic health outcomes including, total cholesterol, high-density lipoprotein (HDL) cholesterol, total cholesterol/HDL ratio, low-density lipoprotein (LDL) cholesterol, non-HDL cholesterol (total cholesterol minus HDL cholesterol), apoB/apoA-1 ratio, IL-6, triglycerides, s-insulin, and glucose. All blood samples were centrifuged immediately and plasma/serum stored frozen prior to being sent to an accredited commercial pathology laboratory for analysis. All blood assays were completed via standard procedures adhering to the NATA guidelines [22].

#### Monitoring of energy intake and expenditure after the protocol

After each lab visit participants wore the validated Sense Wear Mini armband [7] around their upper arm for 48 h and kept a food diary to determine energy expenditure (EE) and energy intake (EI), respectively. The food diary was weighed. Energy intake was derived using the FoodWorks version 7 professional nutritional analysis package [49]. The data was entered into FoodWorks by the senior author (AP) after consultation with a dietician. Food substitutions were recorded for consistency. Where food items were not available e.g. home cooked meals, parents provided the recipe and a new item was created.

### Analysis

#### Randomization and sample size

Participants were randomized by an independent person using a computerised random number generator. This allowed simple random allocation to complete either condition ‘A’ (typical) or ‘B’ (reduced) for Visit 2 (see Fig. 1) and the alternate condition for Visit 3. It was not possible to fully blind the participants as they were able to observe the difference between the conditions during their third visit to the lab.

Cardiometabolic and cognitive outcomes were both primary outcomes of this study. The absence of research investigating the impact of sitting time on cognitive outcomes prevented the calculation of a sample size relating to cognition. Due to the absence of studies investigating the impact of sitting time on cardiometabolic outcomes in healthy adolescents the following process was used. Power and sample size calculations were based on the changes in cardiometabolic outcomes reported in previously conducted studies (change in glucose iAUC of 10% (SD16%), insulin iAUC of 12% (SD20%), and triglyceride iAUC of 34% (SD54%) [17, 52]. Assuming an alpha level of 0.05 and within sample correlations between 0.5 and 0.8 sample sizes were estimated using PROC POWER in SAS V9.4 (SAS Cary NC), the required sample size to obtain a power of 0.80 ranged from 11 to 24 subjects.

### Statistical analyses

Descriptive statistics were calculated on all pre and post measures for each condition. Data were analyzed in IBM SPSS (V21, IBM Corporation Armonk NY) using linear mixed models. Variables that did not meet normality assumptions were log transformed prior to analysis. An extreme outlier (for S-insulin) was removed from the analysis. Effect sizes were included to demonstrate the magnitude of the difference between the means of the two conditions, as effect sizes act independently of sample size [50]. Standardized effect sizes were calculated on complete cases (based on a t-test) and calculated from means and standard deviations using the typical sitting condition as the denominator. Effect sizes of approximately 0.2, 0.5 and 0.8 are considered small, medium and large, respectively [13]. Paired t-tests were conducted on the SenseWear Mini armband data and energy intake following both conditions.

## Results

The rate and sequence of participation throughout the study is shown in Fig. 1. Between May and September 2014, 19 potential participants were assessed for eligibility. Eighteen met the inclusion criteria, attended the familiarisation visit (Visit 1) and consented to participate. Seven participants were classified as overweight or obese using body fat reference curves for children [34]. Participant characteristics at baseline are shown in Table 1. Protocol compliance for both conditions was assessed by direct observation and written records, participants did not deviate from the protocol in either condition.

**Table 1:** Characteristics of Study Participants

	All (*n* = 18)	Participants with complete data (*n* = 14)	Participants with incomplete data (*n* = 4)
Female (% number)	7	5	2
Male (% number)	11	9	2
Age (years)	13.5 ± 0.9	13.4 ± 1.0	13.7 ± 0.8
Height (cm)	161.7 ± 10.0	162.3 ± 11.0	159.6 ± 5.9
Weight (kg)	55.8 ± 14.3	55.5 ± 15.5	56.8 ± 11.1
BMI (kg/m^2^)	21.1 ± 3.9	20.8 ± 3.8	22.3 ± 4.7
Body Fat (%)	23.0 ± 7.6	21.5 ± 5.6	28.1 ± 12.1
Overweight/obese (%)	7 (38.9%)	6	1
Blood Pressure- systolic/diastolic (mm Hg)	115.2/69.7 ± 13.8/7.6	114.1/71.4± 13.44/7.1	118.8/63.8± 16.4/6.9
Resting Heart Rate (bpm	78.1 ± 13.6	77.2 ± 8.2	82.5 ± 37.3

Each individual consumed the same food items during both conditions (average energy intake 4762.6 kJ ± 1596.8 kJ).

Data from four participants were incomplete as the nurse/phlebotomist was unable to draw blood from these participants during Visit 2 and additional data were missing due to processing problems at the pathology lab. As a result, cardiometabolic data were available for 14 participants for the apoB/apoA-1 ratio, 13 complete sets of data were available for total cholesterol, LDL cholesterol, HDL cholesterol, Non-HDLC, triglycerides and glucose and 10 complete sets of data were available for IL6. In addition one outlier was removed during the analysis for LDL cholesterol and IL6 (see Table 2).

**Table 2:** Condition and effects of experimental conditions (reduced sitting versus typical) on cardiometabolic outcomes (mean **+/−** SD) of total study participants (*n* = 18)

Cardiometabolic health indicator	‘Typical’ school day			‘Reduced sitting’ school day			Condition effect		
	Pre	Post	Difference (pre-post)	Pre	Post	Difference (pre-post)	Difference (reduced -typical)	P- value	Effect Size**
Figural Intersection task (Cognitive function)^d^	6.06 ± 0.10	5.89 ± 1.71	−0.17 ± 0.15	5.71 ± 1.53	6.18 ± 1.47	0.47 ± 1.231	0.64 ± 0.15	0.149	0.54
ApolipoproteinA1/ ApolipoproteinB (g/L)^b^	0.52 ± 0.20	0.51 ± 0.20	−0.01 ± 0.03	0.52 ± 0.16	0.49 ± 0.15	−0.03 ± 0.03	−0.02 ± 0.03	0.027	−0.67
Interleukin-6 (fg/ml)^c^	453.71 ± 433.60	1010.53 ± 683.35	556.82 ± 776.23	1945.58 ± 2597.66	2556.97 ± 4185.09	611.39 ± 2410.06	−54.56 ± 2750.40	0.227	0.03
Total cholesterol (mmol/L) ^a^	4.36 ± 1.08	4.34 ± 1.08	−0.01 ± 0.23	4.40 ± 0.82	4.20 ± 0.84	−0.20 ± 0.29	−0.19 ± 0.27	0.283	−0.71
Total cholesterol/HDL ratio (mmol/L)^a^	3.14 ± 1.19	3.32 ± 1.29	0.18 ± 0.23	3.00 ± 0.90	3.44 ± 1.04	0.44 ± 0.68	0.25 ± 0.53	0.251	0.51
HDL-cholesterol (mmol/L) ^a^	1.46 ± 0.41	1.39 ± 0.39	−0.07 ± 0.09	1.52 ± 0.36	1.22 ± 0.44	−0.30 ± 0.50	−0.23 ± 0.50	0.117	−0.66
LDL-cholesterol (mmol/L) ^a^	2.46 ± 1.07	2.34 ± 1.13	−0.12 ± 0.19	2.52 ± 0.83	2.44 ± 0.95	−0.08 ± 0.47	0.04 ± 0.43	0.065	0.12
Non-HDLC (mmol/L) ^a^	2.82 ± 1.08	2.90 ± 1.07	0.08 ± 0.18	2.86 ± 0.85	2.95 ± 0.91	0.09 ± 0.39	−0.01 ± 0.39	0.901	0.03
Triglycerides (mmol/L) ^a^	0.77 ± 0.33	1.21 ± 0.73	0.44 ± 0.45	0.72 ± 0.21	1.12 ± 0.49	0.40 ± 0.45	0.05 ± 0.19	0.718	0.09
Fasting glucose (mmol/L) ^a^	5.23 ± 0.39	4.92 ± 0.51	−0.31 ± 0.32	5.22 ± 0.48	4.95 ± 0.59	−0.27 ± 0.58	0.04 ± 0.77	0.813	−0.09
S-insulin (mU/L) ^a^	14.22 ± 12.70	28.90 ± 33.78	14.68 ± 21.74	10.90 ± 3.24	24.92 ± 16.78	14.02 ± 16.47	0.65 ± 12.25	0.913	0.03
Systolic BP (mmHg)*	115.15 ± 13.76	109.78 ± 11.13	−5.39 ± 10.826	117 ± 13.75	112.72 ± 13.57	−4.28 ± 15.56	1.11 ± 13.40	0.801	0.08
Diastolic BP (mmHg)*	69.67 ± 7.57	65.61 ± 8.29	−4.06 ± 2.6	72.28 ± 10.94	70.78 ± 10.27	−1.50 ± 13.64	2.56 ± 12.40	0.490	0.26

* ln transformed prior to analysis

^ P values for interleukin-6 and LDL-cholesterol were 0.968 and 0.071 respectively with removal of single extreme outlying value

**Effect sizes were calculated on complete cases only for the differences between mean effects

a n = 13

b n = 14

c n = 10

^d^ *n* = 17

A comparison of demographic data for participants who did not have complete sets of blood data compared to those that had complete sets of blood data (Table 1) found there was no significant difference in the scores for age (*M ± SD)* = 13.4 ± 1.0vs (*M ± SD)* = 13.7 ± 0.8, *t* (18) = 0.16, *p* = 0.25, BMI (*M ± SD)* = 20.8 ± 3.8 vs (*M ± SD)* = 22.3 *±* 4.7, t (18) = 0.68, *p* = 0.9, and body fat percentage (*M ± SD)* = 21.5 *±* 5.6) vs (*M ± SD)* = 28.1 *±* 12.1, *t* (18) = 0.71, *p* = 0.18.

### Cognitive function

Differences in mental attention capacity for pre and post typical and reduced-sitting school day are shown in Table 2. Mental attention capacity declined slightly during the typical school day, but increased in the reduced sitting day. The pre to post difference between the two conditions, while not statistically significant, nonetheless had a medium effect size (*d* = 0.54).

### Cardiometabolic outcomes

The cardiometabolic health outcomes for the total sample pre and post typical and reduced sitting school day are also shown in Table 2. The pre to post difference between the two conditions was statistically significant for apoB/apoA-1 ratio and there was a medium effect size in the hypothesized direction for this outcome (*d* = −0.67) and for total cholesterol (*d* = −0.71). There were also medium effect sizes in the non-hypothesized direction for HDL cholesterol; and total cholesterol/HDL ratio. The differences between the reduced and typical school day for glucose, insulin, blood pressure, body fat and BMI were small and not statistically significant (see Table 2).

### Monitoring of energy intake and expenditure

Participant mean energy intake whilst undergoing both trial protocols were 5337.68 ± 1778.83 kJ, mean carbohydrates were 186.77 ± 52.88 g and mean total fat 38.14 ± 18.85 g.

During the 48 h period following the protocol, there was no difference in the total energy expenditure (using Sensewear devices) between the ‘typical’ (M = 10,134.8 kJ ± 48.7 kJ) and ‘reduced sitting’ school day (M = 11,276.4 kJ ± 3458.5 kJ) in the 48 h after each condition (t [14] = −1.06, *P* = 0.31). There was no difference in energy intake (kilojoules) (using food diaries) between the ‘typical’ (M = 15,037.9 kJ ± 7021.5 kJ) and ‘reduced sitting’ school day (M = 14,942.6 kJ ± 3820.0 kJ) in the 48 h after each condition (t [10] = −0.06, *P* = 0.95), indicating that there was no compensation.

## Discussion

This is the first study to examine the difference between adolescent cardiometabolic and cognitive outcomes during a simulated ‘typical’ and ‘reduced’ sitting school day. The only cardio-metabolic outcome exhibiting a significant improvement in pre to post change was apoB/apoA-1 ratio. Several outcomes demonstrated favourable improvements with medium effect sizes for: total cholesterol, HDL cholesterol, total cholesterol/HDL cholesterol ratio and the apoB/apoA-1 ratio. There were no differences for glucose, insulin, blood pressure, body fat or BMI. A medium effect size indicating improvements in outcomes were also observed for cognitive function.

Previous experimental studies investigating the impact of reducing sedentary behavior on cardiometabolic outcomes in adolescents have produced mixed results [6, 43, 46]. Saunders et al. [46] there were no significant differences in participants insulin, glucose, triglycerides, HDL and LDL cholesterol AUC. Ross et al. [43] only assessed post prandial triglycerides with no significant differences in area under the concentration-time curve, however the findings indicated for 8 of the 12 participants triglyceride concentrations remained high during the day spent in 6 h of sitting. Belcher et al. [6] found significantly lower insulin AUC, C-peptide AUC, glucose AUC and free fatty acid concentrations as a result interrupting 3 h of sitting every 30 min with MVPA. The results of the current study may have differed from previous studies for several reasons. First, previous studies used a different dose of physical activity to break up sitting time [6, 43, 46]. For example Saunders et al. trial included three conditions: 8 unbroken hours of sitting, 8 h of sitting broken every 20 min with 2 min of light walking and finally 8 h of sitting broken every 20 min with 2 min of light walking as well as 2 × 20 min periods of MVPA [46]. Belcher et al. interrupted 3 h of sitting with 3 min of MVPA every 30 min [6], while Ross et al. broke up 6 h of sitting every 30 min with 4 min of moderate physical activity [43]. Differences in the dose and type of physical activity and duration of sedentary time may impact lipoprotein lipase (LPL) activity influencing triglyceride and glucose uptake to differing extents, as reduced stimulation of weight bearing muscles results in reduced uptake of these markers [23]. Second the current study measured cardio-metabolic biomarkers at the beginning and end of the simulated school day, whereas previous studies [6, 43, 46] measured postprandial responses at regular intervals across the experimental period. In addition, the post-test blood measures in the current study were not true fasting samples due to the duration between the midday meal and the final blood draw. Whilst evidence indicates that a fasting duration of 3 h is sufficient to achieve comparable blood glucose levels between fasting and non-fasting states in adults [36], the study design limited the opportunity for an equivalent fasting state for the post-intervention phase. In the study by Ross et al. [43], participants consumed a high fat diet prior to the experimental protocol whilst in the other studies participants consumed a balanced diet. Ross et al. [43], blood measures were taken using finger puncture whilst the other studies used venous blood. When comparing the findings mean values for adolescent cholesterol are higher using finger prick as opposed to venous blood [4].

There is a growing body of evidence indicating that apoB/apoA-1 is a powerful biomarker of future cardiovascular disease as it impacts lipid metabolism, and is a marker of inflammation possessing both anti-oxidant and anti-inflammatory effects. In the current study, differences between the conditions resulted in a medium effect size (*d =* 0.54) which were statistically significant for the apoB/apoA-1 ratio [53, 55]. Although to date there have been no experimental studies investigating the link between interruptions to sedentary time and apoB/apoA-1 ratios, a study of 18 healthy 19 to 23 year olds demonstrated statistically significant reductions in apoB/apoA-1 ratios after replacing sitting time with walking or standing [18]. As suggested by Hamilton et al. (2007), non-exercise activity thermogenesis (NEAT) is a large part of total energy expenditure. Muscular contractions that occur when standing or in very light physical activity may inhibit unhealthy molecular signals contributing to metabolic diseases [24].

Keeping in mind the findings in the current study reflect the transition from a fasting to a postprandial state, the reduced sitting day condition had a medium effect on total cholesterol (d = −0.71), HDL cholesterol (*d* = −0.66) and total cholesterol/HDL ratio (*d* = 0.51). Studies show that high total blood cholesterol increases the risk of coronary heart disease and some types of stroke [38]. Early identification and control of high cholesterol in youth has been found to reduce the risk of cardiovascular disease into adulthood [21]. While there are no other experimental adolescent studies reporting an effect for reduced sitting time on total cholesterol, a 9-month prospective uncontrolled trial of overweight and obese office workers observed significant reductions in total cholesterol following the introduction of treadmill work stations [27].

HDL cholesterol has a positive effect on health as it transports lipids away from the heart back to the liver [31]. Prior to the commencement of the study we hypothesised that the intervention would result in improvements in HDL cholesterol [33]. Medium effects sizes for HDL cholesterol (d = −0.66) and total cholesterol/HDL ratio (d = 0.51) in the current study were not in the anticipated direction, however, they are consistent with previous evidence. Two recent reviews of adult studies designed to assess the effects of exercise interventions on cholesterol levels and lipid profiles suggest increases in aerobic exercise (through increases in intensity or duration) and calorific expenditure resulted in improved HDL cholesterol levels [31, 51]. As HDL seems to be related to MVPA and the current study aimed to increase LPA this may explain the lack of effect on HDL cholesterol in this instance. Another potential explanation is that postprandial changes in HDL cholesterol are often inversely associated with changes in triglyceride concentrations [14] (Table 2). In addition, a systematic review of adult interventions designed to clarify the role of LPA on cardiovascular risk factors and markers in adults determined that all studies except one found no significant changes in HDL cholesterol [5]. Although many of the studies were of low or fair quality, recognising a need for further studies.

The total cholesterol/HDL ratio is a good predictor of cardiovascular health [35]. Lower ratios of total cholesterol/HDL indicate lower risk of heart disease, thus research often aims to reduce this risk factor [26, 39]. The findings in the current study, however, were in the non-hypothesised direction. As previously explained, given that total cholesterol decreased and HDL cholesterol also decreased it is not surprising that the total cholesterol/HDL cholesterol ratio would increase.

Whilst evidence indicates that physical activity is positively associated with academic performance among adolescents [20], there is a dearth of research investigating the association between sedentary behaviour and cognitive functioning in adolescents [11]. Recent findings indicate that reducing sedentary time produces positive academic outcomes in adolescents [15] and classrooms with stand-biased desks may have beneficial effects on academic engagement [16]. Medium effect sizes for cognition in the current study indicate that mental attention capacity (a causal component underlying the development of working memory) declined after the typical school day and increased after the reduced sitting day. The size of the difference between the typical and reduced sitting day for mental-attentional capacity has important implications for reductions in sitting through the school setting as it is equivalent to 6 months’ improvement in effective mental-attentional capacity [41]. Rather than sustained, structural changes in cognitive function (i.e., changes in the underlying capacity constraints of working memory), these changes are likely functional (e.g., temporary increases in the effective capacity of working memory, due to increased focus, sustained attention, less distraction from task-irrelevant aspects of a situation). Functional changes in cognition are nevertheless interesting and important for development and academic reasons. For instance, research on Cognitive Load Theory has demonstrated the learning advantages of optimising the focus and deployment of working memory (i.e., increasing attentional capacity focused on learning-relevant information and processes) [29]. As such, even temporary improvements in functional working memory capacity can yield academic and developmental benefits in those situations in which it is optimised. This suggests the potential for acute enhancements to cognitive function (but perhaps also sustained benefits with a longer intervention) as a result of a reduced sitting day in the school environment. It also suggests the possibility of broader academic benefits, given research establishing a link between mental-attentional (working memory) capacity and non-verbal/fluid intelligence and academic ability (e.g., math, reading) [25]. It is therefore possible that simple adjustments to the school classroom and outdoor environment can have acute (and possibly longer-term) effects on cognitive function in young people.

Reducing sitting time by approximately 50% during the school day in the current study did not result in a compensatory reduction in energy expenditure and increase in energy intake among the participants in the 48-h period post intervention. These findings are consistent with another within person comparisons investigating compensatory reductions in energy expenditure in adolescents. Saunders and colleagues [45] exposed children and adolescents to three different experimental conditions involving prolonged and interrupted breaks in sedentary time and found that the participants did not compensate in the 24 h post experiment by increasing food intake or physical activity levels when sedentary time was reduced or interrupted. While these findings support the notion that compensation does not occur over the short term, longer experimental trials are needed to further investigate these findings.

The current study has some strengths and limitations which should be acknowledged. The study findings may be limited by its sample size, potentially affecting the opportunity to detect significant associations for some outcomes. Secondly, the study design did not incorporate multiple blood draws potentially improving the presentation of blood outcomes and comparisons with other studies. Further, data indicating participant maturation was not collected; pubertal maturation has biochemical and physiological effects during adolescents. Finally, a large number of statistical tests were performed without adjustment for multiple comparisons; the results should be interpreted in this context. There were several strengths in this study. The design of this study is a strength in terms of ecological validity [46] as is the first randomized controlled trial investigating the acute effects of reducing adolescent sitting in a setting which replicates the school environment. Further, it considered the impact of sitting time on adolescent cognition, not previously reported. Finally it assessed novel cardiometabolic markers (apoB/apoA-1 ratio and IL-6), not previously considered in this context.

## Conclusions

This study is the first to explore acute differences in cognitive function and cardiometabolic health outcomes in adolescents when comparing traditional and reduced sitting time during a school day. While acknowledging study limitations, the findings show potential to improve adolescent cardiometabolic and cognitive outcomes. There were significant improvements in adolescents’ apoB/apoA-1 ratio, and medium effect sizes for total cholesterol, HDL cholesterol and total cholesterol/HDL ratio. Cognitive function results showed the equivalent of a 6 month improvement in effective mental-attentional capacity. Future research should focus on larger randomized controlled trials with extensive follow-up periods in participants at greater risk of cardiometabolic abnormalities to evaluate whether the acute effects on cognitive function and cardiometabolic health are maintained when habitual sitting time is reduced.

## Additional files

Additional file 1: Condition A: A ‘typical’ school day schedule. Description: A table demonstrating the protocol used to guide participants through the first condition: a ‘typical’ school day. (DOCX 24 kb)

Additional file 2: Condition B: A ‘reduced sitting’ school day schedule. Description: A table demonstrating the protocol used to guide participants through the first condition: a ‘reduced sitting’ school day. (DOCX 12 kb)

Additional file 3: Comparison of total time allocated to sedentary time and physical activity for both experimental protocols. Description: Two tables providing a comparison of the total time spent in sedentary time and physical activity for Condition A (typical school day) and Condition B (reduced sitting school day). (DOCX 14 kb)

## Abbreviations

BMI: Body mass index
CI: Confidence intervals
EE: Energy expenditure
EI: Energy intake
FIT: Figural intersections task
HDL: High-density lipoprotein
iAUC: Increase in area under the curve
IL6: Interleukin 6
LDL: Low-density lipoprotein
MVPA: Moderate-to-vigorous physical activity
NATA: National Association of Testing Authorities, Australia
NEAT: Non-exercise activity thermogenesis
Non-HDLC: Non high-density lipoprotein cholesterol
PKU: Phenylkentonuria
SD: Standard deviation

## References

[1] Alloway TP, Alloway RG (2010). Investigating the predictive roles of working memory and IQ in academic attainment. J Exp Child Psychol.

[2] Altenburg TM, Rotteveel J, Dunstan DW, Salmon J, Chinapaw MJM (2013). The effect of interrupting prolonged sitting time with short, hourly, moderate-intensity cycling bouts on cardiometabolic risk factors in healthy, young adults. J Appl Physiol.

[3] Balagopal P, George D, Patton N (2005). Lifestyle-only intervention attenuates the inflammatory state associated with obesity: a randomized controlled study in adolescents. J Pediatr.

[4] Barrett SC, Huffman FG, Johnson P (2011). Validation of finger-prick testing of fasting blood glucose, Total cholesterol, and HbA1c in adolescents. Point of Care.

[5] Batacan RB, Duncan MJ, Dalbo VJ, Tucker PS, Fenning AS (2015). Effects of light intensity activity on CVD risk factors: a systematic review of intervention studies. Biomed Res Int.

[6] Belcher BR, Berrigan D, Papachristopoulou A (2015). Effects of interrupting Children's sedentary behaviors with activity on metabolic function: a randomized trial. J Clin Endocrinol Metab.

[7] BodyMedia Temple Healthcare Pty Ltd. SenseWear. Mittagong Australia 2014. http://www.templehealthcare.com.au//. Accessed 9 Nov 2015.

[8] Carnevale Schianca GP, Pedrazzoli R, Onolfo S (2011). ApoB/apoA-I ratio is better than LDL-C in detecting cardiovascular risk. Nutr Metab Cardiovasc Dis.

[9] Carson V, Cliff DP, Janssen X, Okely A (2013). Longitudinal levels and bouts of sedentary behaviour among adolescent girls. BMC Pediatr.

[10] Chaput J-P, Borghese M, Saunders TJ (2013). Combined associations between moderate to vigorous physical activity and sedentary behaviour with Cardiometabolic risk factors in children. Can J Diabetes.

[11] Cliff D, Hesketh K, Vella S (2016). Objectively measured sedentary behaviour and health and development in children and adolescents: systematic review and meta-analysis. Obes Rev.

[12] Cliff DP, Jones RA, Burrows TL (2014). Volumes and bouts of sedentary behavior and physical activity: associations with cardiometabolic health in obese children. Obesity.

[13] Cohen J (1988). Statistical power analysis of the behavioural sciences.

[14] Cohn JS, McNamara JR, Cohn SD, Ordovas JM, Schaefer EJ (1988). Postprandial plasma lipoprotein changes in human subjects of different ages. J Lipid Res.

[15] Corder K, Atkin AJ, Bamber DJ (2015). Revising on the run or studying on the sofa: prospective associations between physical activity, sedentary behaviour, and exam results in British adolescents. Int J Behav Nutr Phys Act.

[16] Dornhecker M, Blake J, Benden M, Zhao H, Wendel M (2015). The effect of stand-biased desks on academic engagement: an exploratory study. Int J Health Promot Educ.

[17] Dunstan DW, Kingwell BA, Larsen R (2012). Breaking up prolonged sitting reduces postprandial glucose and insulin responses. Diabetes Care.

[18] Duvivier BMFM, Schaper NC, Bremers MA (2013). Minimal intensity physical activity (standing and walking) of longer duration improves insulin action and plasma lipids more than shorter periods of moderate to vigorous exercise (cycling) in sedentary subjects when energy expenditure is comparable. PLoS One.

[19] Ekelund U, Luan J, Sherar L, Esliger D, Griew P, Cooper A (2012). Moderate to vigorous physical activity and sedentary time and Cardiometabolic risk factors in children and adolescents. JAMA.

[20] Esteban-Cornejo I, Tejero-Gonzalez CM, Sallis JF, Veiga OL (2015). Physical activity and cognition in adolescents: a systematic review. J Sci Med Sport.

[21] Expert Panel on Integrated Guidelines for Cardiovascular Health and Risk Reduction in Children and Adolescents, National Heart, Lung, and Blood Institute (2011). Expert panel on integrated guidelines for cardiovascular health and risk reduction in children and adolescents: summary report. Pediatrics.

[22] Friedewald WT, Levy RI, Fredrickson DS (1972). Estimation of the concentration of low-density lipoprotein cholesterol in plasma, without use of the preparative ultracentrifuge. Clin Chem.

[23] Hamilton MT, Hamilton DG, Zderic TW (2004). Exercise physiology versus inactivity physiology: an essential concept for understanding lipoprotein lipase regulation. Exerc Sport Sci Rev.

[24] Hamilton MT, Hamilton DG, Zderic TW (2007). Role of low energy expenditure and sitting in obesity, metabolic syndrome, type 2 diabetes, and cardiovascular disease. Diabetes.

[25] Howard S, Johnson J, Pascual-Leone J (2013). Measurement of mental attention: assessing a cognitive component underlying performance on standardized intelligence tests. Psychol Test Assess Model.

[26] Iwani NAKZ, Jalaludin MY, Zin RMWM (2017). Triglyceride to HDL-C Ratio is Associated with Insulin Resistance in Overweight and Obese Children. Sci Rep.

[27] John D, Thompson DL, Raynor H, Bielak K, Rider B, Bassett DR (2011). Treadmill workstations: a worksite physical activity intervention in overweight and obese office workers. J Phys Act Health.

[28] Kane M, Conway A, Hambrick D, Engle R, Conway R, Kane M, Miyake A, Towse J (2007). Variation in working memory capacity as variation in executive attention and control. Variation in working memory.

[29] Kirschner PA, Ayres P, Chandler P (2011). Contemporary cognitive load theory research: the good, the bad and the ugly. Comput Hum Behav.

[30] Kwon S, Burns T, Levy S, Janz K (2012). Breaks in sedentary time during childhood and adolescence: Iowa bone development study. Med Sci Sports Exerc.

[31] Mann S, Beedie C, Jimenez A (2014). Differential effects of aerobic exercise, resistance training and combined exercise modalities on cholesterol and the lipid profile: review. Synth Recommend Sports Med.

[32] Martinez-Gomez D, Eisenmann, JC, Healy, GN, et al. Sedentary behaviors and emerging Cardiometabolic biomarkers in adolescents. J Pediatr. 2012; 160(1):104–110.e2. 10.1016/j.jpeds.2011.06.037 21839464

[33] Martínez-Gómez D, Eisenmann JC, Gómez-Martínez S, Veses A, Marcos A, Veiga OL (2010). Sedentary behavior, adiposity, and cardiovascular risk factors in adolescents. The AFINOS study. Rev Esp Cardiol.

[34] McCarthy HD, Cole TJ, Fry T, Jebb SA, Prentice AM (2006). Body fat reference curves for children. Int J Obes.

[35] Millán J, Pintó X, Muñoz A (2009). Lipoprotein ratios: physiological significance and clinical usefulness in cardiovascular prevention. Vasc Health Risk Manag.

[36] Moebus S, Göres L, Lösch C, Jöckel K-H (2011). Impact of time since last caloric intake on blood glucose levels. Eur J Epidemiol.

[37] Moher D, Hopewell S, Schulz k (2010). CONSORT 2010 explanation and elaboration: updated guidelines for reporting parallel group randomised trials. J Clin Epidemiol.

[38] Murray CJL, Lauer JA, Hutubessy RCW (2003). Effectiveness and costs of interventions to lower systolic blood pressure and cholesterol: a global and regional analysis on reduction of cardiovascular-disease risk. Lancet.

[39] Pacifico L, Bonci E, Andreoli G (2014). Association of serum triglyceride-to-HDL cholesterol ratio with carotid artery intima-media thickness, insulin resistance and nonalcoholic fatty liver disease in children and adolescents. Nutr Metab Cardiovasc Dis.

[40] Pascual-Leone J, Baillargeon R (1994). Developmental measurement of mental attention. Int J Behav Dev.

[41] Pascual-Leone J, Johnson, J. A developmental theory of mental attention: its application to measurement and task analysis. In: P Barrouillet Gaillard, V, editors. Cognitive development and working memory: a dialogue between neo-Piagetian theories and cognitive approaches. New York: Psychology Press Ltd; 2011. p. 13–46.

[42] Ridley K, Ainsworth BE, Olds TS (2008). Development of a compendium of energy expenditures for youth. Int J Behav Nutr Phys Act.

[43] Ross K, Hinckson E, Zinn C (2015). Effect of intermittent sitting time on acute postprandial lipemia in children. J Clin Trans Endocrinol.

[44] Saunders T, Larouche R, Colley R, Tremblay M (2015). Acute sedentary behaviour and markers of Cardiometabolic risk: a systematic review of intervention studies. J Nutr Metab.

[45] Saunders TJ, Chaput J-P, Goldfield GS, Colley RC, Kenny GP, Doucet E (2014). Children and youth do not compensate for an imposed bout of prolonged sitting by reducing subsequent food intake or increasing physical activity levels: a randomised cross-over study. Br J Nutr.

[46] Saunders TJ, Chaput J-P, Goldfield GS (2013). Prolonged sitting and markers of cardiometabolic disease risk in children and youth: a randomized crossover study. Metabolism.

[47] Saunders TJ, Tremblay MS, Mathieu M-È (2013). Associations of sedentary behavior, sedentary bouts and breaks in sedentary time with Cardiometabolic risk in children with a family history of obesity. Plos One.

[48] Sisson S, Gibson A, Short K (2013). Light activity following a meal and postprandial cardiometabolic risk in adolescents. Pediatr Exerc Sci.

[49] Software X. Foodworks 8. Australia 2016. https://www.xyris.com.au/. Accessed 14 Apr 2016.

[50] Sullivan GM, Feinn R (2012). Using effect size—or why the P value is not enough. J Grad Med Educ.

[51] Tambalis K, Panagiotakos DB, Kavouras SA, Sidossis LS (2009). Responses of blood lipids to aerobic, resistance, and combined aerobic with resistance exercise training: a systematic review of current evidence. Angiology.

[52] Thorp AA, Kingwell BA, Sethi P, Hammond L, Owen N, Dunstan DW (2014). Alternating bouts of sitting and standing attenuate postprandial glucose responses. Med Sci Sports Exerc.

[53] Van Linthout S, Spillmann F, Riad A (2008). Human Apolipoprotein A-I gene transfer reduces the development of experimental diabetic Cardiomyopathy. Circulation.

[54] Voss MW, Carr LJ, Clark R, Weng T (2014). Revenge of the “sit” II: does lifestyle impact neuronal and cognitive health through distinct mechanisms associated with sedentary behavior and physical activity?. Ment Health and Phys Act.

[55] Yusuf S, Hawken S, Ôunpuu s (2004). Effect of potentially modifiable risk factors associated with myocardial infarction in 52 countries (the INTERHEART study): case-control study. Lancet.

