# Supplementary material for: Acute effects of reducing sitting time in adolescents: a randomized cross-over study
Source: BMC Public Health. 2017 Aug 15;17:657. doi: 10.1186/s12889-017-4660-6 (PMC5558656; doi:10.1186/s12889-017-4660-6)
Supplement: Supplementary file 1 — Condition A: A ‘typical’ school day schedule. Description: A table demonstrating the protocol used to guide participants through the first condition: a ‘typical’ school day. (DOCX 24 kb) [file 12889_2017_4660_MOESM1_ESM.docx]

**Supplementary file 1.** Condition A: A ‘typical’ school day schedule

| **Running**  **Time** | **Start Time** | **Class** | **Task and posture** | **Finish time** | **Calorimeter activity** | **Comment** | **Time of comment** |
| --- | --- | --- | --- | --- | --- | --- | --- |
|  | 09:00 |  | Commence day | 09:00 | Participant to make a distinct jump in the air to indicate beginning of protocol on monitors |  |  |
|  | 9.00 | *Home Room* | Stand (LPA)  (2 minutes) | 9.02 | Ipad activity - personal choice, sway hips side to side, wind desk down |  |  |
| 2 min | 9.02 |  | Sitting task  (8 minutes) | 9.10 | Ipad activity- personal choice. Ensure participant remains seated. |  |  |
| 10 min | 09:10 | *Period 1*  *English* | Walk to class (LPA)  (4 minutes) | 09:14 | Walk on treadmill (2.0 km/hr) |  |  |
| 14 min | 09:14 |  | Sitting task  (20 minutes) | 09:34 | FIT test |  |  |
| 34 min | 09:34 |  | Stand (LPA)  (2 minutes) | 09:36 | Wind desk up, swing left arm 10 times, swing right arm 10 times, wind desk down |  |  |
| 36 min | 09:36 |  | Sitting task  (20 minutes) | 09:56 | FIT test, if finished early, play Ipad activity of choice sitting down |  |  |
| 56 min | 09:56 |  | Stand (LPA)  (3 minutes) | 09:59 | Hand in test, stretch neck and arms |  |  |
| 59 min | 09:59 | *Period 2*  *PE Class* | Change class (LPA)  (4 minutes) | 10:03 | Walk slow pace on treadmill (2.0 km/hr), wind desk down |  |  |
| 1.03 hr | 10:03 |  | Sitting task  (5 minutes) | 10:08 | Wind desk down, sitting, find pulse and record twice for 30 seconds - write on paper at desk |  |  |
| 1.08 hr | 10:08 |  | Stand/walk (LPA)  (3 minutes) | 10:11 | Throwing game using a bucket and ping pong balls- record number of goals on white board |  |  |
| 1.11 hr | 10:11 |  | Stand/walk (LPA)  (3 minutes) | 10:14 | Quoits on the wall - record number of points on white board |  |  |
| 1.14 hr | 10:14 |  | Moderate to Vigorous Physical activity (MVPA)  (5 minutes) | 10:19 | Walk on treadmill (5.0- 6.0 km/hr) option to listen music from Ipad or personal Ipod |  |  |
| 1.19 hr | 10:19 |  | Stand/walk (LPA)  (2 minutes) | 10:21 | Standing, find pulse and record twice for 30 seconds - write on whiteboard |  |  |
| 1.21 hr | 10:21 |  | MVPA  (5 minutes) | 10:26 | Walk on treadmill (5.0- 6.0 km/hr) option to listen music from Ipad or personal Ipod |  |  |
| 1.26 hr | 10:26 |  | Stand/walk (LPA)  (5 minutes) | 10:31 | Wii-game of choice |  |  |
| 1.31 hr | 10:31 |  | MVPA  (5 minutes) | 10:36 | Walk on treadmill (5.0- 6.0 km/hr) option to listen music from Ipad or personal Ipod |  |  |
| 1.36 hr | 10:36 |  | Stand/walk (LPA)  (5 minutes) | 10:41 | Wii- game of choice |  |  |
| 1.41 hr | 10:41 |  | MVPA  (5 minutes) | 10:46 | Walk on treadmill (5.0- 6.0 km/hr) option to listen music from Ipad or personal Ipod, ease down to slower pace |  |  |
| 1.46 hr | 10:46 | *Recess* | Walk to recess & Toilet break (LPA)  (6 minutes) | 10:52 | Walk slow pace on treadmill (2.0 km/hr) (3 minutes)  Unpack lunch from bag, toilet break |  |  |
| 1.52 hr | 10:52 |  | Sitting task  (10 minutes) | 11:02 | Eat recess while playing a game on the Ipad-game of choice |  |  |
| 2.02 hr | 11:02 |  | MPA  (5 minutes) | 11:07 | Walk on treadmill (3.0- 4.0 km/hr) option to listen music from Ipad or personal Ipod |  |  |
| 2.07 hr | 11:07 |  | LPA (start moving to class)  (3 minutes) | 11:10 | Ease off treadmill slowly, after 1 minute, pack up bags and put rubbish in the bin and tidy up area, have a sip of water. Then go back to treadmill |  |  |
| 2.10 hr | 11:10 | *Period 3*  *Science* | Walk to class  (4 minutes) | 11:14 | Walk slow pace on treadmill (2.0 km/hr) |  |  |
| 2.14 hr | 11:14 |  | Sitting task  (17 minutes) | 11:31 | Build 3 boats – listen to instructions and make the boats |  |  |
| 2.31 hr | 11:31 |  | Standing task (LPA)  (10 minutes) | 11:41 | Wind desk up, stand to complete the boats and test them in the water bowl, whilst swaying hips side to side, then wind desk down |  |  |
| 2.41 hr | 11:41 |  | Sitting task  (11 minutes) | 11:52 | Science based Ipad game |  |  |
| 2.52 hr | 11:52 |  | Standing task  (6 minutes) | 11:58 | Wind desk up, continue science Ipad game, whilst marching on the spot |  |  |
| 2.58 hr | 11:58 | *Period 4*  *Art* | Walk to class  (4 minutes) | 12:02 | Walk slow pace on treadmill (2.0 km/hr), ease off, then wind desk down, bring art resources to desk |  |  |
| 3.02 hr | 12:02 |  | Sitting task  (43 minutes) | 12:45 | Get resources out of bag sitting, and commence drawing/painting (can look at Ipad for ideas). Ensure participant remains seated throughout.  Ideas:   - Draw you playing your favourite sport - Place large blobs of paint on paper, fold in half and combine colours - Paint by numbers - Origami |  |  |
| 3.45 hr | 12:45 |  | Stand/walk (LPA)  (2 minutes) | 12:47 | Stand up, participant to write their name on their artwork and attach it to the wall using bluetac. Then pack up all art resources and clean any mess made |  |  |
| 3.47 hr | 12:47 | *Lunch* | Walk to lunch & Toilet break LPA  (6 minutes) | 12:53 | Walk slow pace on treadmill (2.0 km/hr) (3 mins), then drink break, toilet break, fetch lunch from hatch |  |  |
| 3.53 hr | 12:53 |  | Sitting task  (10 minutes) | 13:03 | Eat lunch completely, then allowed to sit and watch something on Ipad |  |  |
| 4.03 hr | 13:03 |  | Stand (LPA)  (5 minutes) | 13:08 | Wind up desk, stand and play Ipad- game of choice, whilst balancing body weight from left to right foot, wind desk down. |  |  |
| 4.08 hr | 13:08 |  | MVPA  (3 minutes) | 13:11 | Walk on treadmill (5.0- 6.0 km/hr), ease off, then wind desk down |  |  |
| 4.11 hr | 13:11 |  | Sitting task  (5 minutes) | 13:16 | Sit and play Ipad- game of choice |  |  |
| 4.16 hr | 13:16 |  | Walk / stand (LPA)  (9 minutes) | 13:25 | Wii- game of choice |  |  |
| 4.25 hr | 13:25 |  | Sitting task  (6 minutes) | 13:31 | Ipad- game or YouTube clips of choice |  |  |
| 4.31 hr | 13:31 | *Period 5*  *Maths* | Walk to class  (4 minutes) | 13:35 | Walk slow pace on treadmill (2.0 km/hr) |  |  |
| 4.35 hr | 13:35 |  | Sitting task  (32 minutes) | 14:07 | Use Ipad to play; ‘Khan Academy’, ‘2048’, Solitaire, Sudoku. |  |  |
| 5.07 hr | 14:07 |  | Standing  (2 minute) | 14:09 | Wind desk up, stand and stretch: [chin tuck](http://www.mayoclinic.com/health/stretching/WL00030&slide=4), [head turn](http://www.mayoclinic.com/health/stretching/WL00030&slide=5), and [side neck stretch](http://www.mayoclinic.com/health/stretching/WL00030&slide=6), wind desk down |  |  |
| 5.09 hr | 14:09 |  | Sitting task  (13 minutes) | 14:22 | Sit and listen to instruction, then play the roll the dice game- rugby or netball |  |  |
| 5.22 hr | 14:22 | *Period 6*  *HSIE* | Walk to class (LPA)  (4 minutes) | 14:26 | Walk on treadmill (2.0 km/hr) |  |  |
| 5.26 hr | 14:26 |  | Sitting task  (20 minutes) | 14:46 | FIT test |  |  |
| 5.46 hr | 14:46 |  | Stand (LPA)  (2 minutes) | 14:48 | Wind desk up, swing left arm 10 times, swing right arm 10 times, wind desk down |  |  |
| 5.48 hr | 14:48 |  | Sitting task  (20 minutes) | 15:08 | FIT test, if finished early play Ipad game of choice |  |  |
| 6.08 hr | 15:08 |  | Stand (LPA)  (2 minutes) | 15:10 | Wind up desk, hand in paper, pack up any resources and tidy area. Finish with some whole body stretches |  |  |
