# Supplementary material for: Acute effects of reducing sitting time in adolescents: a randomized cross-over study
Source: BMC Public Health. 2017 Aug 15;17:657. doi: 10.1186/s12889-017-4660-6 (PMC5558656; doi:10.1186/s12889-017-4660-6)
Supplement: Supplementary file 2 — Condition B: A ‘reduced sitting’ school day schedule. Description: A table demonstrating the protocol used to guide participants through the first condition: a ‘reduced sitting’ school day. (DOCX 12 kb) [file 12889_2017_4660_MOESM2_ESM.docx]

| **Condition B: A ‘reduced sitting’ school day schedule** | **Start time** | **Class** | **Task** | **Finish time** | **Calorimeter Room Activity** | **Comment** | **Time of comment** |
| --- | --- | --- | --- | --- | --- | --- | --- |
|  | 09:00 |  | Commence day | 09:00 | Participant to make a distinct jump in the air to indicate beginning of protocol on monitors |  |  |
|  | 09:00 | *Home Room* | Stand (LPA)  (5 minutes) | 09:05 | White board slam game |  |  |
| 5 min | 09:05 |  | Stand (LPA)  (5 minutes) | 09:10 | Play Wii game of choice. Ensure participant does not sit down. |  |  |
| 10 min | 09:10 | *Period 1*  *English* | Walk to class (LPA)  (4 minutes) | 09:14 | Walk on treadmill (2.0 km/hr) |  |  |
| 14 min | 09:14 |  | Sitting task  (20 minutes) | 09:34 | FIT test |  |  |
| 34 min | 09:34 |  | Stand (LPA)  (2 minutes) | 09:36 | Wind desk up, swing left arm 10 times, swing right arm 10 times and wind desk down. |  |  |
| 36 min | 09:36 |  | Sitting task  (20 minutes) | 09:56 | FIT test, if finished early, play Ipad activity of choice |  |  |
| 56 min | 09:56 |  | Stand (LPA)  (3 minutes) | 09:59 | Hand in test, then play Ipad activity- whilst marching on the spot |  |  |
| 59 min | 09:59 | *Period 2:*  *PE Class* | Walk to class (LPA)  (4 minutes) | 10:03 | Walk on treadmill (2.0 km/hr) |  |  |
| 1.03 hr | 10:03 |  | Sitting task  (2 minutes) | 10:05 | Practice finding pulse on neck and wrist, and give instructions for Ping Pong ball in bucket game |  |  |
| 1.05 hr | 10:05 |  | Stand/move about (LPA)  (6 minutes) | 10:11 | Ping pong game x 2, walk to whiteboard and write how many balls got in the bucket |  |  |
| 1.11 hr | 10:11 |  | Moderate to Vigorous Physical activity (MVPA)  (5 minutes) | 10:16 | Walk on treadmill (5.0- 6.0 km/hr) option to listen music from Ipad or personal Ipod |  |  |
| 1.16 hr | 10:16 |  | Stand/move about (LPA)  (5 minutes) | 10:21 | Quoits on the wall, add up score once all on there and write on whiteboard |  |  |
| 1.21 hr | 10:21 |  | MVPA  (5 minutes) | 10:26 | Walk/run on treadmill (5.0- 6.0 km/hr) option to listen music from Ipad or personal Ipod |  |  |
| 1.26 hr | 10:26 |  | Stand/walk (LPA)  (5 minutes) | 10:31 | Play Wii game of choice |  |  |
| 1.31 hr | 10:31 |  | MVPA  (5 minutes) | 10:36 | Walk/run on treadmill (5.0- 6.0 km/hr) option to listen music from Ipad or personal Ipod |  |  |
| 1.36 hr | 10:36 |  | Stand/walk (LPA)  (5 minutes) | 10:41 | Play Ping Pong game x3, write up scores on whiteboard and compare |  |  |
| 1.41 hr | 10:41 |  | MVPA  (5 minutes) | 10:46 | Walk/run on treadmill (5.0- 6.0 km/hr) option to listen music from Ipad or personal Ipod |  |  |
| 1.46 hr | 10:46 |  | Stand/walk (LPA)  (2 minutes) | 10:48 | Slowly ease off, then work through body stretches |  |  |
| 1.48 hr | 10:48 | *Recess* | Walk to recess and toilet break (LPA)  (6 minutes) | 10:54 | Walk on treadmill (2.0 km/hr) (3 mins), then water break, toilet break and fetch recess from hatch |  |  |
| 1.54 hr | 10:54 |  | Stand (LPA)  (10 minutes) | 11:04 | Whilst slightly swaying hips side to side stand at desk to eat recess. Try to encourage the participant to eat slowly. After completely eaten, play Ipad, marching on the spot |  |  |
| 2.04 hr | 11:04 |  | MVPA  (5 minutes) | 11:09 | Walk/run on treadmill (5.0- 6.0km/h) option to listen music from Ipad or personal Ipod |  |  |
| 2.09 hr | 11:09 |  | Stand/ walk (LPA)  (3 minutes) | 11:12 | Play Wii game of choice |  |  |
| 2.12 hr | 11:12 |  | Start moving to class (LPA)  (2 minutes) | 11:14 | Pack up bag, rubbish in bin, tidy up |  |  |
| 2.14 hr | 11:14 | *Period 5*  *Art* | Walk to class (LPA)  (4 minutes) | 11:18 | Walk on treadmill (2.0 km/hr) |  |  |
| 2.18 hr | 11:18 |  | Stand (LPA)  (5 minutes) | 11:23 | Unpack all art resources needed for one artwork, listen to instruction for drawing and painting artwork to be completed, wind down desk |  |  |
| 2.23 hr | 11:23 |  | Sitting task  (10 minutes) | 11:33 | Paint/draw conduct first art activity – detailed self portrait |  |  |
| 2.33 hr | 11:33 |  | Stand (LPA)  (7 minutes) | 11:40 | Wind desk up, continue activity. If finished early think of your best friend and paint/draw them. |  |  |
| 2.40 hr | 11:40 |  | Stand (LPA)  (5 minutes) | 11:45 | Unpack other resources needed for one artwork, listen to instruction (with plain paper draw a large circle, cut it out, fold in half, make small cuts out of sides and middle, open it is a snowflake! Make several all different sizes and blu tak around room) , start if ready |  |  |
| 2.45 hr | 11:45 |  | Stand (LPA)  (15 minutes) | 12:00 | Conduct second art activity, every minute participant to squat. |  |  |
| 3.00 hr | 12:00 |  | Stand (LPA)  (2 minutes) | 12:02 | Pack up all resources, place artworks on wall, rubbish in the bin, tidy up area |  |  |
| 3.02 hr | 12:02 | *Period 4*  *HSIE* | Walk to class (LPA)  (4 minutes) | 12:06 | Walk on treadmill (2.0 km/hr) |  |  |
| 3.06 hr | 12:06 |  | Stand (LPA)  (5 minutes) | 12:11 | Read through latest National Geographic Magazine/or local Newspaper. Squat to the ground after each minute, wind desk down |  |  |
| 3.11 hr | 12:11 |  | Sitting task  (10 minutes) | 12:21 | Watch Horrible Histories on Ipad, then pause |  |  |
| 3.21 hr | 12:21 |  | Stand (LPA)  (9 minutes) | 12:30 | Wind desk up, continue watching Horrible Histories on Ipad, then pause, wind desk down |  |  |
| 3.30 hr | 12:30 |  | Sitting task  (10 minutes) | 12:40 | Continue watching Horrible Histories on Ipad |  |  |
| 3.40 hr | 12:40 |  | Stand (LPA)  (2 minutes) | 12:42 | Work through stretches of the body; neck, arms, hamstrings, side body |  |  |
| 3.42 hr | 12:42 |  | Sitting task  (5 minutes) | 12:47 | Fill in questionnaire on Horrible histories episode |  |  |
| 3.47 hr | 12:47 |  | Stand (LPA)  (3 minutes) | 12:50 | Wind up desk and name countries for participant to point out on world map, pack up Ipad/other resources, walk to treadmill |  |  |
| 3.50 hr | 12:50 | *Lunch* | Walk to lunch and toilet break (LPA)  (6 minutes) | 12:56 | Walk on treadmill (2.0 km/hr) (3 mins), then drink break and toilet break |  |  |
| 3.56 hr | 12:56 |  | Stand (LPA)  (10 minutes) | 13:06 | Stand at desk to eat lunch. After completely eaten, play Ipad, swaying hips side to side |  |  |
| 4.06 hr | 13:06 |  | Walk/stand (LPA)  (5 minutes) | 13:11 | Wii game of choice, walk to treadmill |  |  |
| 4.11 hr | 13:11 |  | MVPA  (3 minutes) | 13:14 | Walk/run on treadmill (5.0- 6.0 km/hr) option to listen music from Ipad or personal Ipod |  |  |
| 4.14 hr | 13:14 |  | Walk/stand (LPA)  (5 min) | 13:19 | Ping pong game x 2, note scores on whiteboard and compare scores to previous |  |  |
| 4.19 hr | 13:19 |  | Walk/stand (LPA)  (4 minutes) | 13:23 | Walk on treadmill (2.0 km/hr) option to listen music from Ipad or personal Ipod |  |  |
| 4.23 hr | 13:23 |  | Walk/stand (LPA)  (6 minutes) | 13:29 | Wii game of choice |  |  |
| 4.29 hr | 13:29 |  | Walk/stand (LPA)  (4 minutes) | 13:33 | Quoits on the wall, add up score once all on there and write on whiteboard, compare to previous |  |  |
| 4.33 hr | 13:33 | *Period 3-*  *Science* | Walk to class (LPA)  (4 minutes) | 13:37 | Walk on treadmill (2.0 km/hr) |  |  |
| 4.37 hr | 13:37 |  | Stand (LPA)  (4 minutes) | 13:41 | Ipad science activity. After each minute squat, so that the quad and calf are 90 degrees |  |  |
| 4.41 hr | 13:41 |  | Continue standing (LPA)  (10 minutes) | 13:51 | Lava lamp activity (perhaps whilst marching on spot) |  |  |
| 4.51 hr | 13:51 |  | Stand (LPA)  (10 minutes) | 14:01 | Ipad science activity, balance weight of body from left foot to right foot |  |  |
| 5.01 hr | 14:01 |  | Continue standing (LPA)  (7 minutes) | 14:08 | Bath salts activity (perhaps whilst making circles with hips) |  |  |
| 5.08 hr | 14:08 |  | Stand (LPA)  (10 minutes) | 14:18 | Get resources out and make the tornado in the bottle, whilst listening to instructions |  |  |
| 5.18 hr | 14:18 |  | Continue standing (LPA)  (4 minutes) | 14:22 | Potato activity (perhaps whilst balancing weight of body from left to right foot) |  |  |
| 5.22 hr | 14:22 | *Period 6*  *Maths* | Walk to class (LPA)  (4 minutes) | 14:26 | Walk on treadmill (2.0 km/hr) |  |  |
| 5.26 hr | 14:26 |  | Sitting task  (20 minutes) | 14:46 | FIT test |  |  |
| 5.46 hr | 14:46 |  | Stand (LPA)  (2 minutes) | 14:48 | Wind desk up, swing left arm 10 times, swing right arm 10 times, and wind desk down. |  |  |
| 5.48 hr | 14:48 |  | Sitting task  (20 minutes) | 15:08 | FIT test, if finished early, play Ipad activity of choice |  |  |
| 6.08 hr | 15:08 |  | Stand (LPA)  (2 minutes) | 15:10 | Wind up desk, hand in paper, pack up any resources and tidy area. Finish with some whole body stretches |  |  |
