# Supplementary material for: Acute effects of reducing sitting time in adolescents: a randomized cross-over study
Source: BMC Public Health. 2017 Aug 15;17:657. doi: 10.1186/s12889-017-4660-6 (PMC5558656; doi:10.1186/s12889-017-4660-6)
Supplement: Supplementary file 3 — Comparison of total time allocated to sedentary time and physical activity for both experimental protocols. Description: Two tables providing a comparison of the total time spent in sedentary time and physical activity for Condition A (typical school day) and Condition B (reduced sitting school day). (DOCX 14 kb) [file 12889_2017_4660_MOESM3_ESM.docx]

**Supplementary file 3.** Comparison of total time allocated to sedentary time and physical activity for both experimental protocols

Condition A: ‘typical’ school day

|  | **Sedentary** | **LPA** | **MVPA** | **Total Time** |
| --- | --- | --- | --- | --- |
| Recess | 10 | 9 | 5 | 24 minutes |
| Lunch | 21 | 21 | 3 | 45 minutes |
| PE Class | 5 | 22 | 20 | 49 minutes |
| Home Room | 8 | 2 | 0 | 10 minutes |
| Other Classes | 196 | 46 | 0 | 242 minutes |
| **Total** | **240 (65%)** | **102 (27.5%)** | **28 (7.5%)** | **370minutes** |

Condition B: ‘reduced’ school day

|  | **Sedentary** | **LPA** | **MVPA** | **Total Time** |
| --- | --- | --- | --- | --- |
| Recess | 0 | 21 | 5 | 26 minutes |
| Lunch | 0 | 40 | 3 | 43 minutes |
| PE Class | 2 | 27 | 20 | 49 minutes |
| Home Room | 0 | 10 | 0 | 10 minutes |
| Other Classes | 115 | 127 | 0 | 242 minutes |
| **Total** | **117 (31.5%)** | **225 (61%)** | **28 (7.5%)** | **370 minutes** |
